# Adjusting iron and vitamin A status in settings of inflammation: a sensitivity analysis of the Biomarkers Reflecting Inflammation and Nutritional Determinants of Anemia (BRINDA) approach

**DOI:** 10.1093/ajcn/nqaa141

**Published:** 2020-08-04

**Authors:** Sorrel M L Namaste, Jiangda Ou, Anne M Williams, Melissa F Young, Emma X Yu, Parminder S Suchdev

**Affiliations:** The DHS Program, ICF, Rockville, MD, USA; Hubert Department of Global Health, Rollins School of Public Health, Emory University, Atlanta, GA, USA; Hubert Department of Global Health, Rollins School of Public Health, Emory University, Atlanta, GA, USA; McKing Consulting Corporation, Atlanta, GA, USA; Hubert Department of Global Health, Rollins School of Public Health, Emory University, Atlanta, GA, USA; Hubert Department of Global Health, Rollins School of Public Health, Emory University, Atlanta, GA, USA; Hubert Department of Global Health, Rollins School of Public Health, Emory University, Atlanta, GA, USA; CDC, Atlanta, GA, USA

**Keywords:** biomarkers, inflammation, iron, meta-analysis, micronutrient, nutritional assessment, vitamin A

## Abstract

**Background:**

Accurate assessment of iron and vitamin A status is needed to inform public health decisions, but most population-level iron and vitamin A biomarkers are independently influenced by inflammation.

**Objectives:**

We aimed to assess the reproducibility of the Biomarkers Reflecting Inflammation and Nutritional Determinants of Anemia (BRINDA) regression approach to adjust iron [ferritin, soluble transferrin receptor (sTfR)] and vitamin A [retinol-binding protein (RBP), retinol] biomarkers for inflammation (α-1-acid glycoprotein and C-reactive protein).

**Methods:**

We conducted a sensitivity analysis comparing unadjusted and adjusted estimates of iron and vitamin A deficiency using the internal-survey regression approach from BRINDA phase 1 (16 surveys in children, 10 surveys in women) and 13 additional surveys for children and women (BRINDA phase 2).

**Results:**

The relations between inflammation and iron or vitamin A biomarkers were statistically significant except for vitamin A biomarkers in women. Heterogeneity of the regression coefficients across surveys was high. Among children, internal-survey adjustments increased the estimated prevalence of depleted iron stores (ferritin <12 µg/L) by a median of 11 percentage points (pp) (24 pp and 9 pp in BRINDA phase 1 and phase 2, respectively), whereas estimates of iron-deficient erythropoiesis (sTfR >8.3 mg/L) decreased by a median of 15 pp (15 pp and 20 pp in BRINDA phase 1 and phase 2, respectively). Vitamin A deficiency (RBP <0.7 µmol/L or retinol <0.7 µmol/L) decreased by a median of 14 pp (18 pp and 8 pp in BRINDA phase 1 and phase 2, respectively) in children. Adjustment for inflammation in women resulted in smaller differences in estimated iron deficiency than in children.

**Conclusions:**

Our findings are consistent with previous BRINDA conclusions that not accounting for inflammation may result in an underestimation of iron deficiency and overestimation of vitamin A deficiency. Research is needed to understand the etiology of the heterogeneity in the regression coefficients before a meta-analyzed regression correction can be considered.

## Introduction

Iron and vitamin A deficiency have substantial negative health consequences for women and children, but characterizing the extent to which they contribute to morbidity and mortality worldwide is hindered by challenges in their assessment (1–4). Under an inflammatory state, the biomarkers commonly used to measure iron [ferritin and soluble transferrin receptor (sTfR)] and vitamin A [retinol and retinol-binding protein (RBP)] in population-based surveys not only reflect iron and vitamin A status but are also influenced by inflammation (5). The concentrations of these biomarkers are altered as part of the body's acute-phase response to infection or injury and are also likely influenced by inflammation through other mechanisms (5–7). Studies have consistently shown ferritin and sTfR concentrations to be transiently elevated, whereas retinol and RBP concentrations are depressed, during the inflammatory process (8,9). These effects can lead to potentially under- or overestimating micronutrient deficiency depending on the biomarker.

Researchers from the Biomarkers Reflecting Inflammation and Nutritional Determinants of Anemia (BRINDA) project published in 2017 a new mathematical adjustment approach, termed “regression correction,” to estimate the population prevalence of iron and vitamin A status in settings with inflammation (10). Adjustments are made by measuring α-1-acid glycoprotein (AGP) and C-reactive protein (CRP), 2 acute-phase proteins that reflect chronic and acute inflammation, respectively (11). A linear regression is used to adjust the concentrations of the iron and vitamin A biomarkers to account for the effects of inflammation along the continuum of inflammation. Since the release of the BRINDA approach, it has been applied for diverse purposes, such as estimating the magnitude and risk factors for micronutrient deficiencies (12, 13), linking micronutrient status with functional outcomes (14), efficacy trials (15), and program evaluations (16).

The BRINDA project used data from 16 surveys from 14 different countries to develop the regression correction approach (17–19). To address questions of reproducibility, in this present study, we conducted a sensitivity analysis comprising the surveys used to develop the approach and 16 additional surveys from 15 countries that have recently been added to the BRINDA project database. Specifically, with the addition of new data, we examined consistencies and differences in the *1*) relation of inflammation (AGP, CRP) with iron (ferritin, sTfR) and vitamin A (retinol, RBP) biomarkers, *2*) external reference value generated to define low inflammation to avoid over-adjustments when applying the regression correction approach, *3*) regression correction approach using regression coefficients from internal survey-specific data, and *4*) regression correction approach using meta-analyzed external regression coefficients derived from BRINDA data (planned but not undertaken given the observed heterogeneity). We hypothesized that relations between inflammation and nutrient biomarkers would be similar and that a harmonized single regression correction would be feasible using additional BRINDA data.

## Methods

A description of the BRINDA project and data sources have previously been reported (www.brinda-nutrition.org) (20). Briefly, nationally or regionally representative nutrition surveys were used that *1*) were conducted after 2004, *2*) targeted preschool-age children 6–59 mo old (referred to as children) or nonpregnant women of reproductive age 15–49 y old (referred to as women), and *3*) measured a biomarker of iron (ferritin or sTfR) or vitamin A status (retinol or RBP) and a biomarker of inflammation (AGP or CRP). Data used to originally develop the regression correction adjustment approach termed “BRINDA phase 1” were harmonized and merged with new data presented in this study termed “BRINDA phase 2.” The procedures used for the central acquisition, conversion, and validation of the BRINDA phase 1 and phase 2 data sets were similar (10). The BRINDA protocol was reviewed by the institutional review boards of the NIH and was deemed to be non-human-subjects research.

In the present analysis, the generation of reference values for inflammation relied on all surveys with either AGP data, CRP data, or both. All other analyses were restricted to surveys that measured both AGP and CRP. This is based on our previous work where we showed that both AGP and CRP need to be measured to fully capture the inflammatory state on statistical grounds (17–19), as well as a biological rationale to fully capture both chronic and acute inflammation as measured by AGP and CRP, respectively (21).

### Laboratory analysis

Venous or capillary blood was collected from children and women, and plasma or serum was stored at −20°C until analysis. The sandwich-ELISA method in the VitMin Laboratory was used in many of the surveys, and the remaining surveys used comparable methodology based on the relatively limited information that was accessible on the individual surveys’ laboratory methods. **Supplemental Table 1** provides information that was available on the laboratory methods for the biomarkers in each survey. We did not have access to information on the individual laboratories’ quality control or quality assurance procedures.

### Case definitions

Cutoffs for the biomarkers were consistent with previously published BRINDA work and WHO recommendations where available (10) and were defined as follows: depleted iron stores (ferritin <12 µg/L in children and <15 µg/L in women), iron-deficient erythropoiesis (sTfR >8.3 mg/L in children and women), vitamin A deficiency (retinol <0.7 µmol/L or RBP <0.7 µmol/L in children), vitamin A insufficiency (retinol <1.05 µmol/L or RBP <1.05 µmol/L in women), and inflammation (AGP >1 g/L or CRP >5 mg/L in children and women). Vitamin A insufficiency rather than deficiency was selected to define prevalence estimates for women because a concentration cutoff of <0.7 μmol/L in women resulted in a prevalence of vitamin A deficiency ≤1% across all surveys (22).

### Statistical analysis

All statistics were calculated with the use of Stata 15 software (StataCorp) and cross-checked with SAS version 9.4 software (SAS Institute). Correlations between AGP and CRP concentrations and of ferritin, sTfR, and retinol or RBP with AGP and CRP concentrations were calculated with Kendall's τ coefficient with the use of the SOMERSD package weighted for complex survey design (23). The Taylor linearization method was used to obtain unbiased estimates that incorporated the sampling weight, strata, and cluster (as applicable) when analyzing individual surveys. A sensitivity analysis was also done ignoring the complex survey design.

We calculated and presented unweighted pooled prevalence estimates for depleted iron stores, iron-deficient erythropoiesis, and vitamin A deficiency (or insufficiency) by AGP and CRP decile. The retinol and RBP data were pooled in the present analysis based on previous findings showing consistent relations between these biomarkers with both AGP and CRP (24). All data were stratified by BRINDA phase 1 surveys, BRINDA phase 2 surveys, and a combination of all surveys.

The prevalence of iron and vitamin A status was estimated without any adjustments to ferritin, sTfR, and retinol or RBP and is referred to as unadjusted estimates, taking into account the complex survey design. We also performed a sensitivity analysis using unweighted data that did not account for the complex survey design. The BRINDA regression correction approach as described elsewhere (10) was used to adjust ferritin, sTfR, and retinol or RBP concentrations. Additional information on the adjustment approach, including statistical code, can be found on the BRINDA website (www.brinda-nutrition.org). Under this approach, internal survey-specific data were used to apply adjustments referred to as the “internal regression correction.” Individual surveys’ complex survey design was accounted for and, again, an unweighted sensitivity analysis was done. Briefly, adjustments were calculated by adjusting for the influence of AGP and CRP as follows:

(1) \begin{eqnarray*} && \rm{Adjusted\; micronutrient\; biomarker}\nonumber\\ &&=\rm{micronutrient\; biomarker} - \beta1(\rm{AGP}_{\rm{obs}} - \rm{AGP}_{\rm{ref}})\nonumber\\ &&- \beta2(\rm{CRP}_{\rm{obs}} - \rm{CRP}_{\rm{ref}}) \end{eqnarray*}

Micronutrient biomarkers in this study refer to ferritin, sTfR, and retinol or RBP. AGP, CRP, ferritin, sTfR, and retinol or RBP were continuous variables and were ln transformed. Some CRP data contained values of 0; thus, a constant value of 0.001 was added to CRP data with values of 0 before applying a ln transformation. This differs from the previous BRINDA results where the survey-specific lowest value was used to replace zeros before applying ln transformations and may result in slightly different estimates from previously published work.

The internal regression correction used β-coefficients derived from each individual survey; β1 is the AGP regression coefficient, whereas β2 is the CRP regression coefficient. A test of multicollinearity between ln AGP and ln CRP was assessed on the basis of a test of tolerance (>0.1) and a variance inflation factor (<5). Consistent with previously published BRINDA work, we did not include CRP in the regression correction models for sTfR because of the “underlying physiologic mechanism wherein elevated CRP prevents the rise of sTfR during the early acute-phase response” (18). The β-coefficients were calculated both with and without accounting for the complex survey design.

“Ref” is the external reference value that is defined as low inflammation and refers to the maximum value of the lowest decile category using BRINDA data. Following the same approach as previously used to generate a reference value, CRP data with values of 0 were replaced with the survey-specific lowest nonzero value before generating the reference value (10). The deciles for the pooled reference value were combined with the use of random-effects meta-analysis whereby the metafor package in R 3.5.0 software (R Foundation for Statistical Computing) was used to obtain a common estimate across data sets. The correction is only applied to individuals with ln-AGP > ln-AGP_ref_, ln-CRP > ln-CRP_ref_, or both. We calculated and compared reference values using pooled results for BRINDA phase 1 surveys, BRINDA phase 2 surveys, and a combination of all surveys.

Unadjusted and adjusted prevalence estimates were compared with the use of McNemar's chi-square statistics; statistical significance was defined as *P *< 0.05.

## Results

### Participant characteristics

**FIGURE 1:**
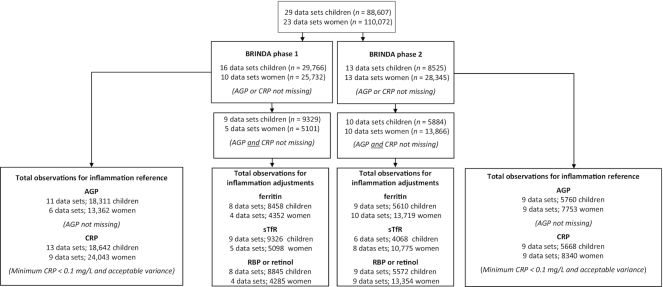
Sample sizes for BRINDA project analyses. The total sample size for BRINDA analyses for the reference value calculations is based on surveys with available AGP or CRP data. The total sample size for BRINDA analyses for the adjustment calculations is based on surveys with available data for both AGP and CRP and either ferritin, sTfR, retinol, or RBP concentrations. Pakistan 2011 is included in BRINDA phase 1 for children but included in BRINDA phase 2 for women. Only AGP data were available in BRINDA phase 1 and CRP data became available in BRINDA phase 2 for women. Thus, Pakistan data (8,261 women) were included in the calculations of the AGP reference for BRINDA phase 1 but are counted under BRINDA phase 2 in this flowchart. AGP, α-1-acid glycoprotein; BRINDA, Biomarkers Reflecting Inflammation and Nutritional Determinants of Anemia; CRP, C-reactive protein; RBP, retinol-binding protein; sTfR, soluble transferrin receptor.

Our study sample for calculating the reference value for AGP and CRP in children was 24,071 and 24,310, respectively. In women, the study sample was 21,115 for AGP and 32,383 for CRP (**Figure 1**). Mongolia 2006 was excluded from the AGP reference value calculation because the variance was near 0. Bangladesh 2012, Burkina Faso 2010, Ecuador 2012, Georgia 2009, and United Kingdom 2008–2014 were excluded from the CRP reference value calculation because the minimum CRP concentration was >0.1 mg/L (limit of detection too high). Analyses examining the relation between micronutrient biomarkers and inflammation and adjustments included surveys that had values for both AGP and CRP and resulted in a total of 17 surveys with ferritin, 15 surveys with sTfR, and 17 surveys with retinol or RBP for children and 14 surveys with ferritin, 13 surveys with sTfR, and 13 surveys with retinol or RBP for women (Figure 1).

Surveys had different inclusion criteria for age in children, resulting in a median age ranging from 8 to 48.1 mo across surveys (overall median age: 30.3 mo). Children were younger in BRINDA phase 1 than in phase 2 (BRINDA phase 1: median, 23.0 mo; range: 8.0–34.0 mo and BRINDA phase 2: median, 36.7 mo; range: 27.0–48.1 mo). In women, the inclusion criterion for age was the same across surveys (15–49 y) with the median ranging from 25.0 to 29.0 y (median: 29.0 y), which was similar across BRINDA phases (data not shown).

**FIGURE 2:**
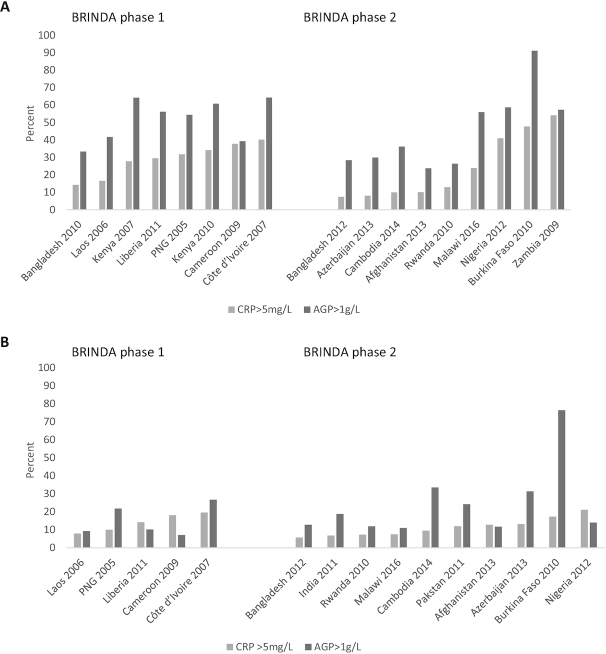
Estimated prevalence of inflammation in children and women, BRINDA project. (A) Estimated prevalence of inflammation for children, (B) estimated prevalence of inflammation for women, with BRINDA phase 1 on the left and BRINDA phase 2 on the right. Estimated prevalence of inflammation defined as CRP >5 mg/L (light gray) and AGP >1 g/L (dark gray), ordered from lowest to highest inflammation amounts based on CRP >5 mg/L. Total sample size in children across surveys is 14,936 and in women 18,817. AGP, α-1-acid glycoprotein; BRINDA, Biomarkers Reflecting Inflammation and Nutritional Determinants of Anemia; CRP, C-reactive protein; PNG, Papua New Guinea.

The prevalence of elevated AGP was higher than the prevalence of elevated CRP in nearly all surveys (**Figure 2**). The median prevalence of any inflammation (AGP concentrations >1 g/L or CRP concentrations >5 mg/L) was 52.8% in children and 20.0% in women. Inflammation was generally lower in the surveys from BRINDA phase 2 in children (median: 38.3% and 57.2% in BRINDA phase 2 and 1, respectively), but not in women (median: 24.8% and 19.2% in BRINDA phase 2 and 1, respectively).

### Correlations between inflammation biomarkers and of iron and vitamin A biomarkers with inflammation

In children, there was a moderate correlation between AGP and CRP concentrations in the positive direction (median: 0.51; range: 0.25–0.63) and the strength was generally similar between the BRINDA phases (**Supplemental Table 2**). In women, the relation was generally weaker than in children, but the patterns were the same (**Supplemental Table 3**).

The correlations of ferritin with the 2 biomarkers of inflammation were moderate, consistently in the positive direction (median: 0.30 for AGP, 0.25 for CRP; range: 0.05–0.44 for both AGP and CRP), and similar between BRINDA phases in children (Supplemental Table 2). This relation was maintained for women, but the strength was weaker and in the negative direction in 1 survey (Rwanda) (Supplemental Table 3).

There was a weak to moderate positive relation between sTfR and AGP in children (AGP: median: 0.18; range: <0.01–0.54), and a weak inconsistent relation between sTfR and CRP in both BRINDA phases (CRP: median: 0.11; range: >−0.01 to 0.21) (Supplemental Table 2). The relation of sTfR with the 2 biomarkers of inflammation among women was similar to that among children in all surveys and by BRINDA phases (Supplemental Table 3).

The correlations of retinol or RBP with the 2 biomarkers of inflammation were moderate and in the negative direction in children, except for Cambodia and Burkina Faso where a positive relation was found (AGP: median: −0.20; range: −0.32 to 0.48; CRP: median: −0.26; range: −0.37 to 0.08) (Supplemental Table 2). The strength of the relation was slightly stronger in BRINDA phase 1 than in phase 2 for AGP and similar between BRINDA phases for CRP (Supplemental Table 2). The relation of retinol or RBP with the 2 biomarkers of inflammation was inconsistent across surveys in women (Supplemental Table 3).

### Relation between iron and vitamin A biomarkers by inflammation deciles

**FIGURE 3:**
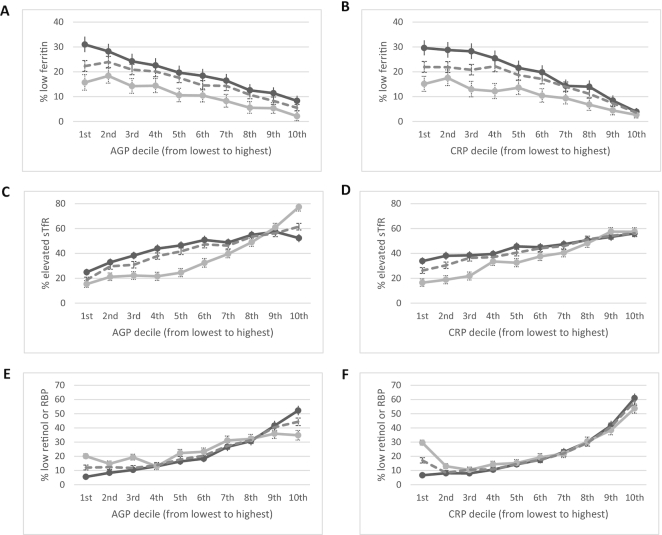
Estimated prevalence [% (95% CI)] of depleted iron stores, iron-deficient erythropoiesis, and vitamin A deficiency by inflammation deciles in children, BRINDA project. Estimated unweighted prevalence of depleted iron stores by AGP deciles (A) and CRP deciles (B), iron-deficient erythropoiesis by AGP deciles (C) and CRP deciles (D), and vitamin A deficiency by AGP deciles (E) and CRP deciles (F) in children. Depleted iron stores defined as ferritin <12 µg/L, iron-deficient erythropoiesis as sTfR >8.3 mg/L, and vitamin A deficiency as either retinol or RBP <0.7 µmol/L. Dashed line, pooled BRINDA phase 1 and 2 data; dark gray line, BRINDA phase 1 data; and light gray line, BRINDA phase 2 data. Sample size: (A, B) 8458 for BRINDA phase 1, 5610 for BRINDA phase 2, and 14,068 for BRINDA pooled; (C, D) 9326 for BRINDA phase 1, 4068 for BRINDA phase 2, and 13,394 for BRINDA pooled; (E, F) 8845 for BRINDA phase 1, 5572 for BRINDA phase 2, and 14,417 for BRINDA pooled. AGP, α-1-acid glycoprotein; BRINDA, Biomarkers Reflecting Inflammation and Nutritional Determinants of Anemia; CRP, C-reactive protein; RBP, retinol-binding protein; sTfR, soluble transferrin receptor.

**FIGURE 4:**
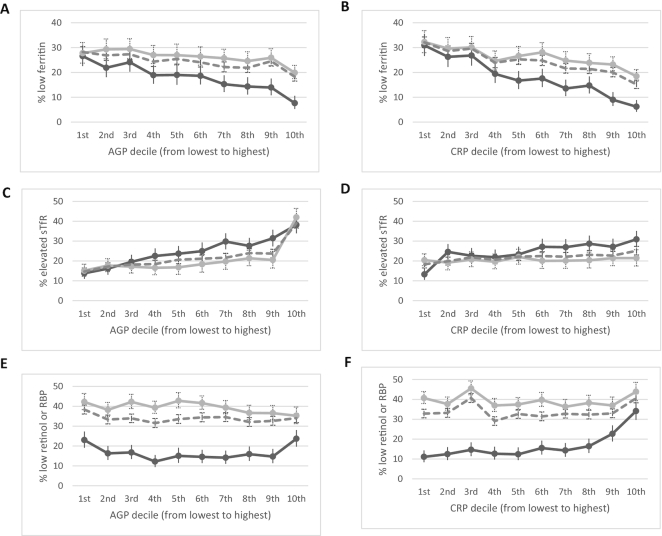
Estimated prevalence [% (95% CI)] of depleted iron stores, iron-deficient erythropoiesis, and vitamin A insufficiency by inflammation deciles in women, BRINDA project. Estimated prevalence of depleted iron stores by AGP deciles (A) and CRP deciles (B), iron-deficient erythropoiesis by AGP deciles (C) and CRP deciles (D), and vitamin A insufficiency by AGP deciles (E) and CRP deciles (F) in women. Depleted iron stores defined as ferritin <15 µg/L, iron-deficient erythropoiesis as sTfR >8.3 mg/L, and vitamin A insufficiency as either retinol or RBP <1.05 µmol/L. Dashed line, pooled BRINDA phase 1 and 2 data; dark gray line, BRINDA phase 1 data; and light gray line, BRINDA phase 2 data. Sample size: (A, B) 4352 for BRINDA phase 1, 13,719 for BRINDA phase 2, and 18,071 for BRINDA pooled; (C, D) 5098 for BRINDA phase 1, 10,775 for BRINDA phase 2, and 15,873 for BRINDA pooled; (E, F) 4285 for BRINDA phase 1, 13,354 for BRINDA phase 2, and 17,639 for BRINDA pooled. AGP, α-1-acid glycoprotein; BRINDA, Biomarkers Reflecting Inflammation and Nutritional Determinants of Anemia; CRP, C-reactive protein; RBP, retinol-binding protein; sTfR, soluble transferrin receptor.

The relation between the estimated prevalence of iron deficiency and inflammation deciles visually appeared to follow a consistently negative direction in children (**Figure 3**) and women (**Figure 4**). The estimated prevalence of depleted iron stores incrementally increased as the concentrations of AGP and CRP decreased and iron-deficient erythropoiesis incrementally decreased as the concentrations of AGP and CRP decreased, except in women where there was little difference in the estimated prevalence of iron-deficient erythropoiesis by CRP concentrations. The estimated prevalence of vitamin A deficiency, whether measured by retinol or by RBP in children, increased with increasing AGP and CRP deciles in children (Figure 3) but was not consistent in women for vitamin A insufficiency (Figure 4).

### Reference values at low amounts of inflammation

The external reference values from BRINDA phase 1 in children were −0.52 ln(g/L) and −2.26 ln(mg/L) for AGP and CRP, respectively. The external reference values from BRINDA phase 1 in women were −0.63 ln(g/L) and −1.83 ln(mg/L) for AGP and CRP, respectively. This equated to 0.59 g/L for AGP and 0.10 mg/L for CRP in children and 0.53 g/L for AGP and 0.16 mg/L for CRP in women. The external reference values from BRINDA phase 2 in children were −0.63 ln(g/L) and −2.37 ln(mg/L) for AGP and CRP, respectively. The external reference values from BRINDA phase 2 in women were −0.67 ln(g/L) and −1.91 ln(mg/L) for AGP and CRP, respectively. This equated to 0.53 g/L for AGP and 0.09 mg/L for CRP in children and 0.51 g/L for AGP and 0.15 mg/L for CRP in women. Given the similar values between phases, reference values from BRINDA phase 1 were used in the regression adjustments.

### Regression correction slopes for ferritin, sTfR, retinol, and RBP

There was no multicollinearity between ln AGP and ln CRP. The slopes from the multiple linear regression with ln AGP and ln CRP as the explanatory variables and the ln-transformed iron and vitamin A biomarkers as the outcome showed substantial variation across surveys (data not shown).

The slopes for ln AGP and ln CRP with ln ferritin as the outcome were nearly all in the positive direction in children (BRINDA phase 1: median: 0.75; range: 0.33–1.23 for AGP and median: 0.13; range: 0.02–0.26 for CRP; BRINDA phase 2: median: 0.67; range: 0.11–1.04 for AGP and median: 0.04; range: −0.01 to 0.18 for CRP) and in women (BRINDA phase 1: median: 0.40; range: 0.16–0.45 for AGP and median: 0.10; range: 0.08–0.11 for CRP; BRINDA phase 2: median: 0.42; range: −0.16 to 0.65 for AGP and median: 0.06; range: −0.04 to 0.16 for CRP) (data not shown).

The slopes for ln AGP with ln sTfR as the outcome were in the positive direction for all surveys in children (BRINDA phase 1: median: 0.33; range: 0.10–0.55; BRINDA phase 2: median: 0.39; range: 0.02–0.76) and women (BRINDA phase 1: median: 0.27; range: 0.18–0.43; BRINDA phase 2: median: 0.26; range: 0.09–0.63) (data not shown).

The slopes for ln AGP and ln CRP with ln retinol or RBP as the outcome were nearly all in the negative direction for all children (BRINDA phase 1: median: −0.14; range: −0.21 to −0.07 for AGP and median: −0.05; range: −0.07 to −0.04 for CRP; BRINDA phase 2: median: −0.06: range: −0.30 to 0.78 for AGP and median: −0.04; range: −0.20 to −0.01 for CRP) (data not shown). β-Coefficients and adjustments to vitamin A biomarkers for inflammation are not presented for women because of the inconsistent relation in retinol and RBP found in this population.

### Unadjusted prevalence estimates of iron and vitamin A deficiency

There was substantial variation in the prevalence of unadjusted depleted iron stores and unadjusted iron-deficient erythropoiesis across surveys in children and women (**Supplemental Tables 4, 5**). The prevalence range of unadjusted depleted iron stores was generally lower in BRINDA phase 2 than in BRINDA phase 1 in children (BRINDA phase 1: median: 17.9; range: 8.0–38.7; BRINDA phase 2: median: 5.4; range: 0–22.0) and higher in BRINDA phase 1 than in BRINDA phase 2 in women (BRINDA phase 1: median: 15.7; range: 12.8–22.7; BRINDA phase 2: median: 10.1; range: 2.7–45.8). Unadjusted iron-deficient erythropoiesis estimates were generally higher in BRINDA phase 2 than in BRINDA phase 1 in children (BRINDA phase 1: median: 35.5; range: 4.1–76.7; BRINDA phase 2: median: 52.3; range: 3.1–85.8). In women, the unadjusted iron-deficient erythropoiesis estimates were generally similar between phases but with a wider range in BRINDA phase 2 (BRINDA phase 1: median: 28.3; range: 5.8–33.8; BRINDA phase 2: median: 29.0; range: 3.0–80.8).

Likewise, there were considerable differences in the prevalence of unadjusted vitamin A deficiency across surveys in children (**Supplemental Table 6**). The prevalence of unadjusted vitamin A deficiency in BRINDA phase 2 generally had similar estimates to BRINDA phase 1 in children although the median was higher in BRINDA phase 1 (BRINDA phase 1: median: 24.4; range: 6.9–29.6; BRINDA phase 2: median: 20.6; range: 8.9–56.6).

### Internal regression correction–adjusted prevalence of iron and vitamin A deficiency

The application of internal regression corrections to ferritin concentrations generally increased the estimated prevalence of depleted iron stores compared with unadjusted estimates (**Table 1**, **Supplemental Tables 7–9**). The converse was found for iron-deficient erythropoiesis and vitamin A deficiency, with adjustments to sTfR concentrations resulting in decreased prevalence estimates and adjustments to retinol or RBP concentrations generally resulting in decreased prevalence estimates (Table 1, Supplemental Tables 7–9).

**TABLE 1:** Summary of changes in the estimated prevalence of iron and vitamin A deficiency with the use of pooled data in children and women, BRINDA project^1^

	Percentage point difference		
	Depleted iron stores	Iron-deficient erythropoiesis	Vitamin A deficiency
Children			
BRINDA phase 1	+24.0 (+7.9, +35.2)	−14.6 (−23.8, −0.8)	−18.0 (−21.5, −6.0)
BRINDA phase 2	+8.6 (+1.1, +13.2)	−19.5 (−70.3, −1.2)	−8.2 (−16.0, −3.4)
BRINDA pooled	+10.5 (+1.1, +35.2)	−15.1 (−70.3, −0.8)	−13.5 (−21.5, −3.4)
Women			
BRINDA phase 1	+9.1 (+3.7, +10.5)	−9.7 (−13.7, −1.9)	NA
BRINDA phase 2	+6.8 (+1.3, +13.3)	−2.6 (−37.7, −1.5)	NA
BRINDA pooled	+7.4 (+1.3, +13.3)	−6.4 (−37.7, −1.5)	NA

1 Values are absolute median differences (ranges) using internal regression correction adjusted for α-1 acid glycoprotein and C-reactive protein compared with unadjusted estimates. Depleted iron stores defined as ferritin <12 µg/L for children and ferritin <15 µg/L for women, iron-deficient erythropoiesis as soluble transferrin receptor >8.3 mg/L for children and women, and vitamin A deficiency as either retinol or retinol-binding protein <0.7 µmol/L for children. Restricted to surveys that were statistically different (*P *< 0.05); for depleted iron stores and iron-deficient erythropoiesis this excluded Rwanda 2010 in children, for vitamin A deficiency this excluded Burkina Faso 2010 in children, and for depleted iron stores this excluded Cambodia 2014 in women. Total sample size for depleted iron stores in children: 13,492 and in women: 17,366; for iron-deficient erythropoiesis in children: 12,818 and in women: 15,873; and for vitamin A deficiency in children: 15,796. BRINDA, Biomarkers Reflecting Inflammation and Nutritional Determinants of Anemia; NA, not applicable.

Among the iron biomarkers, adjustments to ferritin concentrations resulted in a greater absolute percentage point (pp) difference from unadjusted estimates in BRINDA phase 1 (pp difference: median: 24.0; range: 7.9–35.2) than in BRINDA phase 2 (pp difference: median: 8.6; range: 1.1–13.2) in children. The difference in adjusted and unadjusted estimates was statistically significant (*P *< 0.05) for all surveys except for Rwanda 2010. In contrast, adjustments to sTfR concentrations resulted in decreased estimates in all surveys and similar absolute median pp differences from unadjusted estimates between the BRINDA phases (BRINDA phase 1: median: −14.6; range: −23.8 to −0.8; BRINDA phase 2: median: −19.5; range: −70.3 to −1.2) in children. The difference was statistically significant (*P *< 0.05) for all surveys except for Rwanda 2010.

In women, there was an increase in the prevalence estimates for depleted iron stores in all surveys and a decrease in the prevalence estimates of iron-deficient erythropoiesis in all surveys. Among the iron biomarkers, adjustments to ferritin concentrations resulted in a similar absolute pp difference from unadjusted estimates in BRINDA phase 1 (pp difference: median: 9.1; range: 3.7–10.5) compared with BRINDA phase 2 (pp difference: median: 6.8; range: 1.3–13.3) in women. The difference was statistically significant (*P *< 0.05) for all surveys except for Cambodia 2014. In contrast, adjustments to sTfR concentrations resulted in greater absolute median pp differences from unadjusted estimates in BRINDA phase 1 (median: −9.7; range: −13.7 to −1.9) than in BRINDA phase 2 (median: −2.6; range: −37.7 to −1.5) in women. The difference was statistically significant (*P *< 0.05) for all surveys.

There was a decrease in estimated vitamin A deficiency based on retinol or RBP concentrations in all surveys in children after applying adjustments, with a greater absolute pp difference in BRINDA phase 1 than in BRINDA phase 2 (BRINDA phase 1: median: −18.0; range: −21.5 to −6.0; BRINDA phase 2: median: −8.2; range: −16.0 to −3.4). The difference was statistically significant (*P *< 0.05) for all surveys except for Burkina Faso 2010.

When comparing weighted regression estimates and unweighted regression estimates, there was no difference in terms of the direction of the estimates for ln AGP and ln CRP. In children, 1 out of 17 was >3% for ferritin; 1 out of 17 was >3% for retinol or RBP; the differences in prevalence were <3% for sTfR (15 surveys). In women, the differences in prevalence after correction for inflammation were <3% for ferritin (14 surveys) and sTfR (13 surveys). 

## Discussion

Using geographically diverse data from ∼15,000 children and ∼18,000 women, we found the association between inflammation and indicators of iron and vitamin A status (e.g., ferritin, sTfR, retinol, or RBP) was generally consistent across the concentration ranges of AGP and CRP, with the exception of vitamin A status in women. These findings suggest that the BRINDA regression correction remains a strategy that can be used to adjust iron in children and women and vitamin A biomarkers in children for inflammation in population surveys. However, there remained heterogeneity in the slopes of inflammatory proteins and micronutrient biomarkers, requiring the use of a survey-specific internal regression correction, rather than a single correction for inflammation across settings.

The overall adjusted prevalence of estimated depleted iron stores (ferritin) was higher by 10.5 pp in children and 7.4 pp in women and estimated iron-deficient erythropoiesis (sTfR) was lower by 15.1 pp in children and 6.4 pp in women. The adjusted prevalence of estimated vitamin A deficiency was lower by 13.5 pp in children. As noted previously, the dampened effect on corrections for the iron biomarkers and inconsistencies in the inflammation–vitamin A relation in women could be related to the lower amounts of inflammation in this population and differences in the underlying cause of inflammation (e.g., obesity) (17–19,24). Restricting the analysis to surveys that had both AGP and CRP may have introduced some selection bias but allowed us to make comparisons between unadjusted and adjusted estimates. These results are comparable with previous work from BRINDA, which supports the reproducibility of the regression correction approach (17–19, 24).

The BRINDA regression correction uses a linear regression model to make adjustments. We found the relation of iron and vitamin A biomarkers with inflammation was consistent across the decile plots, and that the overall strength of the association was similar to our previous work (17–19,24). Recent data from longitudinal studies support the changes we have observed in micronutrient biomarker concentrations related to the acute-phase proteins. For example, in a norovirus challenge study of healthy adults, ferritin concentrations increased, and retinol and RBP concentrations decreased, in response to temporary elevations in AGP and CRP (25). No changes in micronutrient biomarkers were observed among uninflamed individuals over time, which further suggests that iron and vitamin A biomarker concentration changes in the inflamed group were temporal and not reflective of underlying nutritional status (25). A study among asymptomatic children in Burkina Faso found that short-term, within-individual changes in ferritin and RBP in relation to AGP and CRP were similar to between-individual cross-sectional differences, supporting the validity of inflammation adjustment approaches in cross-sectional surveys (26). However, accounting for repeated measures yielded inflammation-adjusted concentrations more similar to the measured (unadjusted) biomarker concentration than did a cross-sectional approach.

Another important finding from this analysis was defining a reference value for AGP and CRP considered to be “normal,” because we demonstrated that micronutrient biomarker concentrations are altered even at concentrations of AGP and CRP below clinically relevant cutoffs. Using geographically diverse data from 22 surveys of ∼24,000 children and ∼40,000 women, we verified that the external reference values for AGP and CRP, originally obtained by taking the lowest decile from the pooled data sets used at the time of the development of the BRINDA approach, were nearly the same when regenerating the external reference value with the additional data in this sensitivity analysis. For settings with higher prevalence of inflammation, such as in individuals clinically ill, an internal reference value for inflammation may need to be calculated to avoid extrapolation outside of the range of data and over-adjustment. Similarly, utilizing a AGP or CRP method with limits of detection >0.5 g/L and >0.1 mg/L for AGP and CRP, respectively, would require an internal reference value.

As was found previously (17–19,24), there was considerable heterogeneity between the AGP and CRP regression coefficients used in the internal regression corrections to adjust for iron and vitamin A deficiency across surveys; given this finding, we did not make adjustments using external pooled regression coefficients. The reason for this variation is still unknown and raises the question of whether this is an issue with the validity of the regression correction methodology itself or the result of other factors. One explanation could be differences among analytical methods or standards changing over time despite the same laboratory or comparable methods being used in this study. Even more likely, natural biological variation is playing an important role in the observed heterogeneity. Studies are needed to better understand the mechanisms for how or if individual or population characteristics, environmental and socioeconomic determinants, types of infections, or other disorders differentially affect inflammation, as well as the relation of inflammation with iron and vitamin A biomarkers. Further, confounding effects of factors beyond inflammation (e.g., genetics, damage to liver cells, cancers, sex-differentials, lactation) may also influence the interpretation of iron or vitamin A biomarkers and were not considered in this study (22,27). Of lesser concern but still of practical importance is that the lack of a universal regression coefficient makes it harder to implement the adjustments, but this has been addressed through the development of macros similar to the WHO child growth standards which can be found at www.brinda-nutrition.org.

The lack of a gold-standard biomarker for iron and vitamin A deficiency that is not influenced by inflammation to compare the BRINDA correction against remains a limitation. In addition, regarding vitamin A biomarkers, we used retinol and RBP interchangeably. Although there were insufficient data to substantiate a 1:1 ratio for retinol:RBP, the relations of retinol and RBP with inflammation were similar, and based on previous BRINDA work, applying regression adjustments did not alter the retinol:RBP ratio (24). Future research to validate the BRINDA regression correction against gold-standard biomarkers (e.g., bone marrow iron and liver reserves of vitamin A) remains a priority. Nevertheless, the fact that the findings from this sensitivity analysis substantiate previous BRINDA work indicates regression adjustments remain an important approach to statistically account for the confounding impact of inflammation on iron and vitamin A biomarkers.

A major strength of this study was the use of many surveys from diverse settings, using the same or similar collection procedures and laboratory methodologies. However, information on the quality control or quality assurance measures during fieldwork and in the laboratory was unavailable for some of the surveys. It was also not possible to distinguish between missing AGP or CRP concentrations and the assay being performed but the concentrations being below the assays’ limits of detection. We assumed that when AGP and CRP concentrations were below the assays’ limits of detection, the data sets included values of 0 or the lowest level of detection. We previously conducted a sensitivity analysis of surveys conducted at a single laboratory with a known CRP limit of detection of 0.5 mg/L; replacing CRP values with 0.25 mg/L yielded similar correlation analyses (28, 29). The absence of detailed information on the standards used in each survey limits our ability to interpret the results.

Accurate data on iron and vitamin A deficiencies provide vital information to countries and global actors. Our findings indicate that there is a clear need to measure biomarkers of inflammation when assessing iron and vitamin A status. Ignoring inflammation likely results in an underestimation of iron deficiency and overestimation of vitamin A deficiency, which, in turn, may lead to poor resource allocation and inaccurate targeting of micronutrient programs.

## Supplementary Material

nqaa141_Supplemental_File

## Notes

Supported by the Bill & Melinda Gates Foundation, CDC, Eunice Kennedy Shriver National Institute of Child Health and Human Development, HarvestPlus, and the United States Agency for International Development.

Supplemental Tables 1–9 are available from the “Supplementary data ” link in the online posting of the article and from the same link in the online table of contents at https://academic.oup.com/ajcn/.

Data described in the article, code book, and analytic code will be made available upon request pending approval from the BRINDA steering committee and country representatives.

Abbreviations used: AGP, α-1-acid glycoprotein; BRINDA, Biomarkers Reflecting Inflammation and Nutritional Determinants of Anemia; CRP, C-reactive protein; pp, percentage point; RBP, retinol-binding protein; sTfR, soluble transferrin receptor.
